# Fear of Movement and Low Self-Efficacy Are Important Barriers in Physical Activity after Renal Transplantation

**DOI:** 10.1371/journal.pone.0147609

**Published:** 2016-02-04

**Authors:** Dorien M. Zelle, Eva Corpeleijn, Gerald Klaassen, Elise Schutte, Gerjan Navis, Stephan J. L. Bakker

**Affiliations:** 1 Department of Nephrology, University of Groningen, University Medical Center Groningen, Groningen, The Netherlands; 2 Department of Epidemiology, University of Groningen, University Medical Center Groningen, Groningen, The Netherlands; Kings College, UNITED KINGDOM

## Abstract

**Background:**

Physical activity (PA) and exercise are commonly used as preventive measures for cardiovascular disease in the general population, and could be effective in the management of post-transplantation cardiovascular risk. PA levels are low after renal transplantation and very few renal transplant recipients (RTR) meet the PA guidelines. Identification of barriers to regular PA is important to identify targets for intervention to improve PA levels after renal transplantation. We investigated fear of movement and physical self-efficacy as barriers to PA in RTR.

**Methods:**

RTR were investigated between 2001–2003. The Tampa Score of Kinesiophobia–Dutch Version (TSK-11) was used to assess fear of movement. Physical self-efficacy was measured with the LIVAS-scale. PA was assessed using validated questionnaires (Tecumseh Occupational Activity Questionnaire and the Minnesota Leisure Time Physical Activity Questionnaire).

**Results:**

A total of 487 RTR (age 51±12 years, 55% men) were studied. Median score [interquartile range] on TSK-11 was 22 [17–26]. Low physical self-efficacy (Exp B:0.41[0.31–0.54], p<0.001) and history of myocardial infarction, transient ischemic attack and cerebrovascular accident (Exp B:1.30[1.03–1.63],p = 0.03) were independent determinants for fear of movement. Fear of movement was associated with lower daily PA, occupational, sports and leisure time PA. Mediation-analysis showed that a large part (73%) of the effect of fear of movement on PA was explained by low physical self-efficacy.

**Conclusions:**

This study was the first to examine fear of movement and self-efficacy in relation to PA in RTR. Fear of movement was associated with a low PA level, and the larger part of this relation was mediated by low physical self-efficacy. Both fear of movement and physical self-efficacy level are important targets for intervention during rehabilitation after renal transplantation.

## Introduction

After transplantation many renal transplant recipients (RTR) are at high cardiovascular disease risk, of which, new onset of diabetes after transplantation, hypertension, and being overweight play an important role [1–3]. Accordingly, the incidence of cardiovascular disease (CVD) in RTR is four to six times higher than in the general population [4,5]. Therefore management of cardiovascular risk factors is of great importance in the post-transplant setting.

Physical activity (PA) and exercise can positively influence blood pressure, lipid profile and insulin sensitivity and is commonly used as a preventive measure for CVD [6–10]. We previously showed that regular PA after transplantation was strongly associated with a lower risk for cardiovascular and all-cause mortality [11]. Promotion of regular PA could be an effective tool in the management of post-transplantation cardiovascular risk. The KDIGO Guideline for the Care of Kidney Transplant Recipients recommends that patients follow a healthy lifestyle, including regular physical activity [12]. However, PA levels remain low after transplantation and few RTR meet the general guideline for regular PA [1,11,13,14]. In this guideline regular physical activity is defined as 30 minutes of moderate PA a day five days per week. This total amount of physical activity can be undertaken in different contexts or domains, which are related to: occupation, active commuting, leisure (recreational activities, household, climbing stairs) and exercise and sports. As some RTR may be hindered by factors related to the transplantation or recovery, like tiredness or an inability to work, it may be that some domains contribute more to the total physical activity level than others. Understanding why some patients succeed in being physically active after renal transplantation and other patients do not may help to identify important barriers to regular PA. To date, it is unclear which determinants hinder PA after renal transplantation. One possibility is that patients are uncertain about their ability to be regularly physically active or fear they may injure their kidney [15,16].

The fear of movement model, refers to anxiety that individuals can experience when engaging in activities that involve bodily movement [17]. Patients with fear of movement tend to avoid PA because it might cause pain or harm [18]. This avoidance behavior can create a vicious cycle of inactivity. Fear of movement is found to be associated with low PA levels in several patient populations [19,20]. Although fear of movement was originally defined for patients with musculoskeletal pain, it may also apply to other patients groups such as RTR [21,22]. Self-efficacy is a well-known predictor of PA, and is regarded an important target when PA is pursued [23–26]. Based on the theory of social learning, self-efficacy refers to an individual’s beliefs about their capability to perform a particular behavior or task [27]. Individuals with high physical self-efficacy are more likely to initiate and persist activities that aid their recovery, like daily walking. The opposite is seen in individuals with low physical self-efficacy [28]. Self-efficacy is influenced by both physiological and emotional states, such as muscle pain, fatigue, mood, stress and fears like fear of movement [25,27]. Therefore, we hypothesize that physical self-efficacy acts as a mediator in the relationship between fear of movement and PA.

In this paper, we assessed fear of movement in a cohort of RTR, and determined the associations with daily PA and the various domains of PA in this population. Furthermore, we investigated physical self-efficacy as a mediator in the relationship between fear of movement and PA.

## Materials and Methods

### Design and Subjects

All RTR with a functioning graft of more than one year were invited to take part in this study. Patients were recruited in an outpatient clinic from 2001 to 2003. The group that did not sign informed consent was comparable with the group that did sign informed consent with respect to age, sex, body mass index, serum creatinine, creatinine clearance, and proteinuria. Patients who had received a combined transplantation (i.e. kidney/pancreas or kidney/liver) were invited to participate as well. In patients with fever or other signs of infection (e.g. complaints of upper respiratory tract infection or urinary tract infection), baseline visits were postponed until symptoms had resolved. Patients with overt congestive heart failure and patients diagnosed with cancer other than cured skin cancer were not considered eligible for the study. A total of 606 out of 847 eligible RTR signed written informed consent. Participants in this study did not receive any specific advice concerning physical activity. Data on fear of movement were available in 487 RTR. Full details on the study design have been previously reported [29]. The Institutional Review Board of the University Medical Center Groningen approved the study protocol (METc 2001/039).

### Renal Transplant Characteristics

The Groningen Renal Transplant Database contains information on all renal transplantations performed at our center since 1968. Relevant transplant characteristics such as age, gender, dialysis duration and date of transplantation were extracted from this database. Information on working situation, smoking and alcohol consumption, and history of myocardial infarction (MI), transient ischemic attack (TIA) and cerebrovascular accident (CVA) were obtained by self-report questionnaire.

### Daily physical activity

PA was estimated using the Tecumseh Occupational Activity Questionnaire (TOAQ) and the Minnesota Leisure Time Physical Activity Questionnaire (MLTPAQ). These questionnaires, completed by interview with trained research assistants, estimate the total amount of PA over the past 12 months. The TOAQ measures frequency, intensity and duration of a maximum of three occupation-related activities within the previous 12 months. Physical activities associated with transportation to work are also included. The TOAQ is an acceptable measure of occupational PA energy expenditure and has been widely used [30,31]. The MLTPAQ measures leisure time physical activities including household activities, climbing stairs and conditioning physical activity like sports, over the previous 12 months. Both questionnaires have been extensively validated in the general population [32,33]. A combination of these two questionnaires was used to estimate daily PA by using metabolic equivalents of task (MET) [34,35]. These questionnaires can be combined, because they measure both intensity and duration of the activity, which allows calculation summary scores in MET minutes per day (MET-min/d). MET-minutes are calculated by multiplying the intensity (indicated by the MET score) and the duration spent on that activity (measured in minutes). The MET-score can be derived from tables (the Compendium of Physical Activities) that indicate the intensity of the activity relative to resting [36]. The combination of these questionnaires cover the whole of physical activities during the day. For example if one would perform one hour of brisk walking per day as a single activity, the total MET-min/d for PA would be 60 x 5 = 300 MET-min/d. Additionally, we assessed how many RTR fulfilled the general PA guideline. According to this guideline, adults should perform 30 minutes of moderate PA per day, for five days per week. Because moderate PA corresponds to a MET-score of 5 (brisk walking or mid-tempo cycling are typical examples), the guidelines correspond to 30x 5 = 150 MET min/ day on at least five days per week. More concise information on measurements of PA was described previously [11].

### Fear of movement

Fear of movement was measured with the Tampa Score for Kinesiophobia-11 (TSK-11) [37]. Each question is answered on a four-point Likert type scale, ranging from “strongly disagree” to “strongly agree.” The total score ranges from 11–44 with a higher score indicating a higher level of fear of movement. We used the Dutch translation whereby the same scoring format was maintained [17]. The TSK-11 is a brief, reliable and valid measure of fear of movement [38]. The TSK-11 was originally designed to measure fear of movement/(re)injury in individuals with pain. For the current study, small adaptations were made to the original TSK-11 for the use in the renal transplant population. In pain specific statements, we replaced the word 'pain' by the word 'health problems". An example of one of these modified questions is item 2: " If I were to overcome it, my *health problems* would increase".

### Physical self-efficacy

Physical self-efficacy was measured with the LIVAS-scale [39]. The LIVAS-scale is a Dutch translation of the Perceived Physical Activity Scale, which is a sub-scale of the Physical Self-Efficacy Scale [40]. Physical self-efficacy was determined by 10 questions asking subjects to evaluate their physical capacities compared to other people of their own age, on a 5 point Likert type scale, with higher scores representing more positive physical self-efficacy beliefs. Items include comparisons on: flexibility, reaction time, overall strength, physical condition, smooth movements, climbing stairs, strength in hands, walking speed, balance, and overall activity. Example: Compared to most people of my age I probably walk (1) "much slower", (2) "somewhat slower", (3) "just as fast", (4) "somewhat faster", (5) "much faster". The LIVAS-scale is a suitable instrument for measuring physical dimensions of self-efficacy with satisfactory internal consistency with a coefficient alpha of 0.8 [39].

### Depression and anxiety

Quantitative information on depression and anxiety was obtained by self-report questionnaire, using the subscales of the Symptom Checklist (SCL-90) [41]. The SCL-90 is designed to measure a broad range of psychological problems and symptoms of psychopathology. We previously used the SCL-90 to determine depression after renal transplantation [42].

### Body composition

Body Mass Index (BMI) was determined as a measure of overall obesity. Waist circumference as a measure of abdominal obesity was assed at the level midway between the lowest rib and the iliac crest. Muscle mass was estimated by 24-hr urinary creatinine excretion as described earlier [43]. Twenty-four hour urinary creatinine excretion is considered a reliable measure of muscle mass even in patients with advanced renal failure, in elderly people, and in patients with wasting [43–46].

### Clinical measurements and Definitions

Blood was drawn after an overnight fasting period, which included no intake of medication. Creatinine clearance was calculated from 24-hour urinary creatinine excretion and serum creatinine. Blood pressure was measured as the average of three automated (Omron M4;Omron Europe B.V., The Netherlands) measurements with one minute intervals after a six minute rest in the supine position. Post transplantation diabetes mellitus at moment of inclusion was defined by fasting plasma glucose concentration ≥ 7.0 mmol/l or use of anti-diabetic medication.

### Statistical Analyses

Data were analyzed with SPSS version 19.0 (SPSS Inc., Chigago, IL). Normally distributed variables were expressed as mean ± SD, whereas skewed distributed variables are given as median with interquartile range, (IQR); percentages were used to summarize categorical variables. Recipient-related characteristics were analyzed separately for the group with below the median score for fear of movement (low fear of movement) and the group above the median score (high fear of movement). Differences between groups were tested for statistical significance with Student’s t-test for normally distributed variables, Mann–Whitney test for skewed distributed variables, and chi-squared test for categorical variables.

Univariate and multivariate logistic-regression analysis were performed including all variables with a p≤0.1, to determine associations with fear of movement scores greater than the median as binary outcome. For these, analysis variables were transformed into z-scores, which results in expression of Exp (B) per standard deviation change. Pearson correlation analysis was used to determine the relationship between fear of movement and physical self-efficacy, total daily PA and domains of PA. Univariate and multivariate linear regression analysis were used to determine the association between fear of movement and total daily PA. Possible mediation by self- efficacy on the association between fear of movement and PA was examined. In mediation analysis, a third variable is included in the model, known as a mediator-variable, whose influence explains how the two variables are related. The Preacher and Hayes method was used to test the magnitude and significance of mediation [47,48]. First, the total effect of fear of movement on PA was estimated using regression analysis. Second, the indirect effect of fear of movement on PA (via self-efficacy) was calculated by computing the product of two coefficients that were obtained with regression analysis of self-efficacy with I) fear of movement and II) PA. Third, significance of the indirect effect was assessed with bias-corrected bootstrap confidence intervals with 2000 repetitions. Finally, the magnitude of mediation was calculated by dividing the coefficient of the indirect effect by the total effect.

## Results

### Fear of movement

A total of 487 RTR were studied (mean age 50.9 ± 12.0;57% men). Median score[IQR] on TSK-11 was 22 [17–26] for the total population. In Table 1, characteristics of RTR and major study variables are shown according to median score group on the TSK-11 (low and high fear of movement). High fear of movement in RTR was related to other psychological factors, including higher depression and anxiety scores whereas physical self-efficacy levels, were much lower. For body composition, we found no differences in adiposity parameters; however muscle mass was significantly lower in RTR with high fear of movement. High fear of movement was related to a lower creatinine clearance and a longer dialysis duration. With regard to socio-economic status, RTR with high fear of movement had paid employment less often. Lastly, RTR with high fear of movement, had a higher prevalence of MI, TIA and CVA historically, and were more often medically unfit for work.

**Table 1:** Differences between patients based on fear of movement.

	Fear of movement		
	≤ Median	> Median	P-value
	N = 259	N = 228	
**General characteristics**			
Age (yrs)	50.4 ± 11.5	51.6 ± 12.5	0.3
Gender (Male), n (%)	147 (56.8)	131 (57.5)	0.9
**Mental condition**			
Self-efficacy, LIVAS score	26 [23–30]	21[17–25]	<0.001
Depression score	20 [17–24]	22 [19–29]	<0.001
**Physical activity**			
Total daily physical activity, (METS)	164.6 [36.9–368.0]	94.7 [17.0–252.2]	<0.001
Anxiety score	12 [10–13]	13 [11–17]	0.002
**Body composition**			
Body mass index (kg/m2)	26.2 ± 4.3	26.1 ± 4.0	0.7
Waist circumference (cm) women	95.0 ± 14.7	93.6 ± 13.9	0.5
Waist circumference (cm) men	98.9 ± 12.0	100.6 ± 12.9	0.2
Muscle mass (24 hr creatinine excretion, mmol/24hr)	12.3 [10.0–14.7]	11.6 [9.3–13.9]	0.02
**Employment status**			
Paid employment, n(%)	109 (42)	61 (27)	<0.001
Medically unfit for work, n (%)	55 (21)	73 (32)	0.007
**Cardiovascular risk**			
History of myocardial infarction, TIA,CVA, n (%)	17 (7)	40 (18)	<0.001
Systolic blood pressure (mmHg)	150.7 ± 21.9	154.8 ± 23.5	0.05
Diastolic blood pressure (mmHg)	89.5 ± 9.9	90.2 ± 10.1	0.5
Post Transplantation Diabetes Mellitus, n (%)	25 (10)	31 (14)	0.2
**Renal function, transplantation**			
Living donor, n (%)	43 (17)	26 (11)	0.1
Time after translpantation	6.01 [2.7–11.1]	6.24 [3.0–11.2]	0.5
Dialysis duration (months)	23 [11–43]	30 [14–53]	0.001
Creatinine clearance (ml/min)	64 [51–81]	57 [44–74]	0.01

Data are represented as mean ± SD, or median [95% CI]. Differences were tested by t test or Kruskal Wallis test for continuous variables and with Chi- square for categorical variables. All biochemical parameters are determined in fasting blood samples

Univariate logistic regression analyses were used to determine the strengths of the variables associated with fear of movement (Table 2). Upon multivariate logistic regression analysis it appeared that history of MI, TIA and CVA and self-efficacy were independently associated with fear of movement. None of the other analyzed variables remained significant.

**Table 2:** Univariate and multivariate logistic regression analysis with fear of movement.

	Univariate		Multivariate	
Z-scores	Beta [95% CI]	P-value	Beta [95% CI]	P-value
Depression score	1.65 [1.32–2.05]	<0.001	0.97 [0.67–1.40]	0.9
Self-efficacy, LIVAS score	0.38 [0.30–0.48]	<0.001	0.41 [0.31–0.54]	<0.001
Anxiety score	1.67 [1.32–2.11]	<0.001	1.37 [0.92–2.03]	0.1
History of MI, TIA,CVA,	1.45 [1.19–1.77]	<0.001	1.30 [1.03–1.63]	0.03
Dialysis duration	1.32 [1.08–1.62]	0.007	1.12 [0.89–1.42]	0.3
Medically unfit for work	1.29 [1.07–1.55]	0.007	0.86 [0.67–1.10]	0.2
Paid employment	0.72 [0.60–0.86]	<0.001	0.84 [0.66–1.07]	0.2
Creatinine clearance	0.76 [0.63–0.91]	0.003	0.90 [0.69–1.18]	0.4
Systolic blood pressure	1.20 [1.00–1.44]	0.05	1.12 [0.89–1.40]	0.3
Muscle mass	0.81[0.67–0.97]	0.02	1.18 [0.90–1.54]	0.9

### Fear of movement and Physical activity

**Fig 1:**
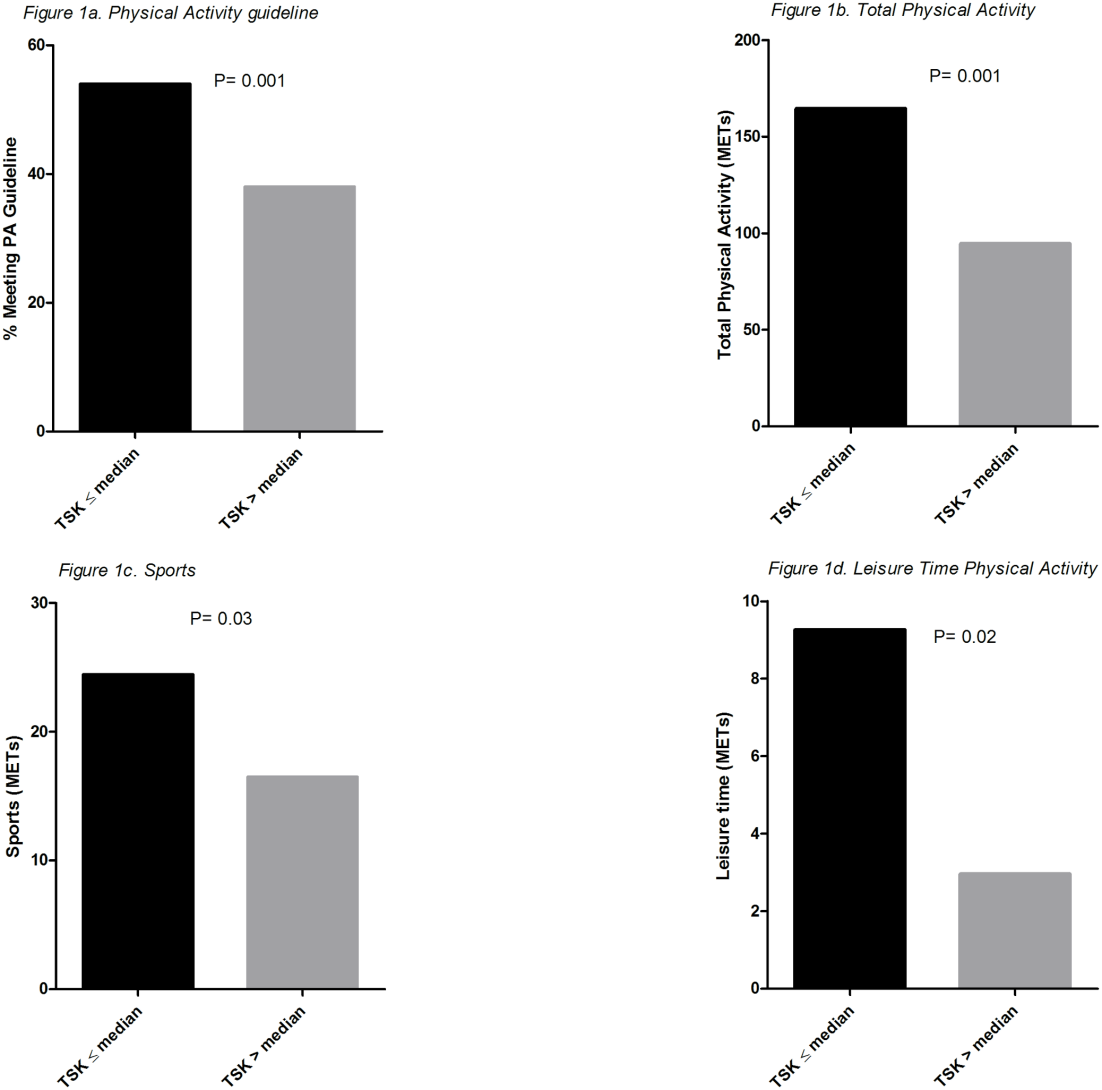
Fear of movement and domains of PA.

Results from the pearson correlation analysis for fear of movement with total daily PA and the various domains of PA are shown in Table 3. We found a significant correlation between fear of movement and total daily PA, consistent with the notion that RTR with higher scores on fear of movement perform less PA. Fear of movement was also associated with occupation, sports and leisure time PA. No associations were found for active commuting, or climbing stairs. With regard to the PA guideline, 54% of the RTR with low fear of movement met the general PA guideline, compared to only 38% of the RTR with high fear of movement. Fig 1 shows that RTR with high fear of movement had significantly lower total daily PA as well as lower scores on sports and leisure time PA. Results of univariate and multivariate linear regression analyses for fear of movement and daily PA level are shown in Table 4. Fear of movement was strongly associated with daily PA (Model 1) in the univariate analysis (Beta = -9.08 [-12.8;-5.3], p<0.001). These associations, slightly weakened after adjustment for age and sex (Model 2). Adjustment for muscle mass, blood pressure, creatinine clearance (Model 3–5) did not significantly change the association. Further adjustment for history of MI, TIA, and CVA (Model 6) weakened the association. The association remained the same after adjustment for time after transplantation (Model 7). After final adjustment for self-efficacy, the association between fear of movement and PA lost significance.

**Table 3:** Pearson correlation analysis (*r*, *P*-value) for factors associated with fear of movement.

	R	P-value
Total daily PA	-0.22	<0.001
Sports	-0.12	0.01
Leisure time PA	-0.12	0.02
Active commuting	-0.05	0.3
Occupation related	-0.16	0.001
Climbing stairs	0.02	0.7

**Table 4:** Linear regression analysis for fear of movement with total daily PA.

Fear of movement	Beta [95% CI]	P-value
Model		
1	-9.08 [-12.8;-5.3]	<0.001
2	-8.63 [-12.1;-5.2]	<0.001
3	-8.04 [-11.5;-4.6]	<0.001
4	-8.05 [-11.5;-4.6]	<0.001
5	-8.06 [-11.5;-4.6]	<0.001
6	-7.63 [-11.1;-4.1]	<0.001
7	-7.63 [-11.2;-4.1]	<0.001
8	-3.83 [-7.8;0.08]	0.06

Model 1: Univariate

Model 2: model 1 + adjustment for age and sex

Model 3: model 2 + adjustment for muscle mass

Model 4: model 3 + adjustment for systolic blood pressure

Model 5: model 4 + adjustment for creatinine clearance

Model 6: model 5 + adjustment for history of MI, TIA,CVA,

Model 7: model 6: +adjustment for time after transplantation

Model 8: model 7 + adjustment for self-efficacy

### Mediation by physical self-efficacy

**Fig 2:**
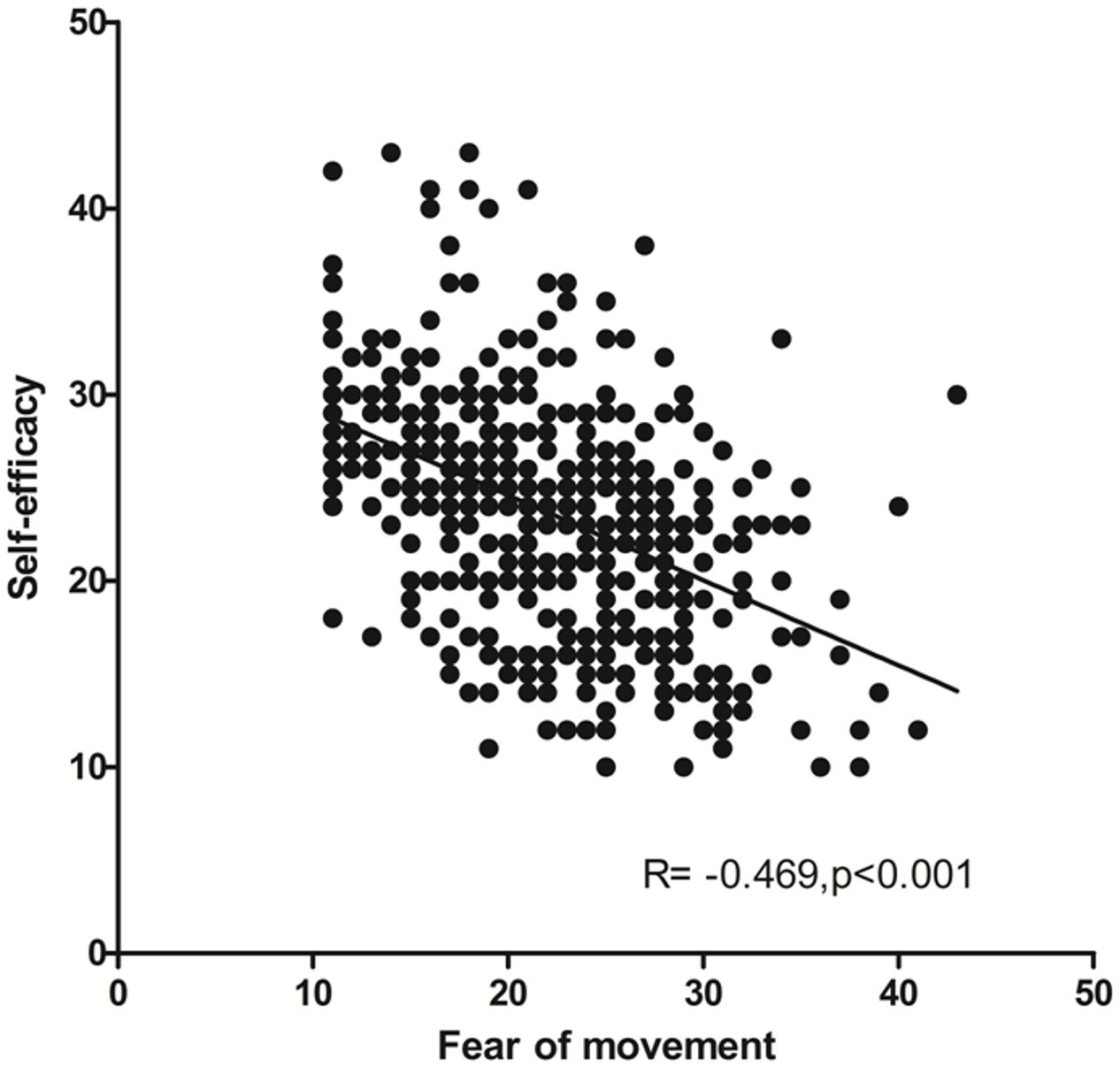
Fear of movement is strongly related to low self-efficacy in RTR.

**Fig 3:**
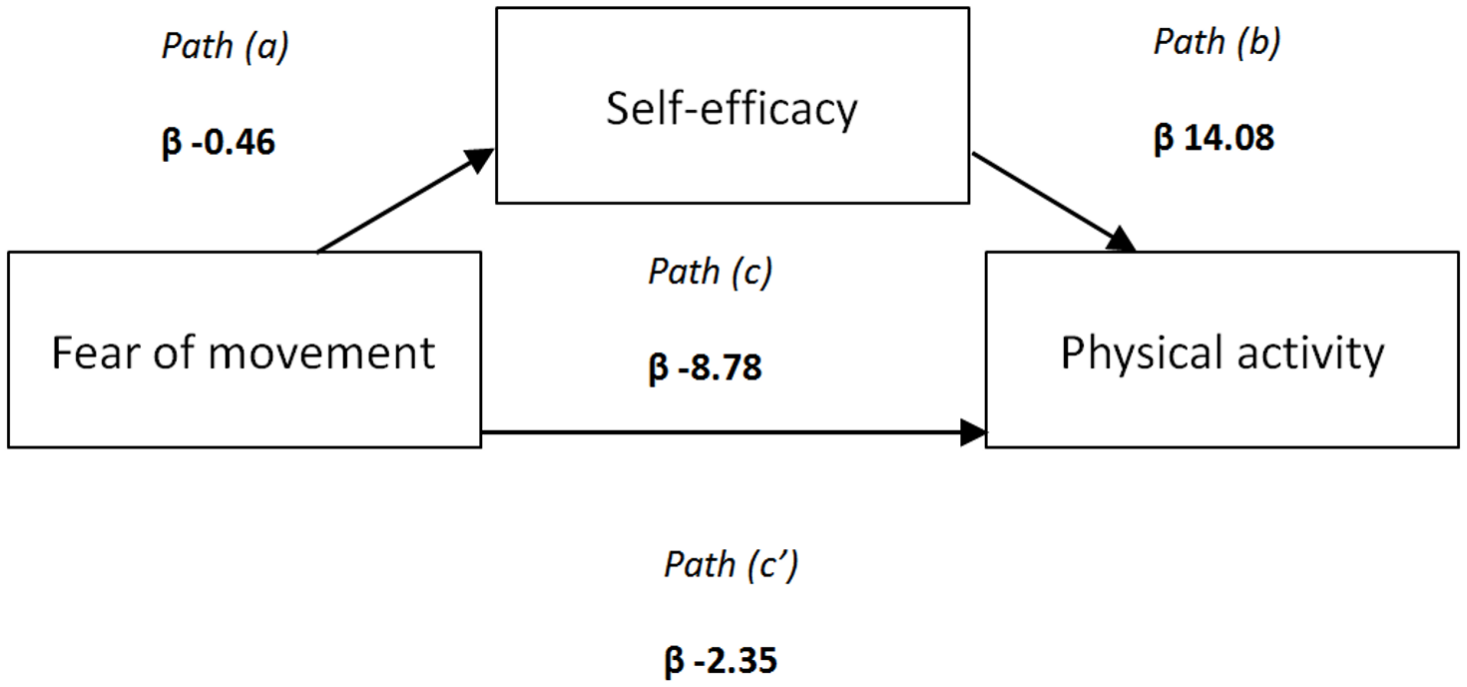
Mediation model of fear of movement and PA through self-efficacy. **Legend:** a, b and c are the standardized regression coefficients between variables. The indirect effect (through self-efficacy) is calculated as a*b. The total effect is a*b+c. The magnitude of mediation is calculated as the indirect effect divided by the total effect.

Fig 2 depicts the relation between fear of movement and self-efficacy. It appears that RTR with a high self-efficacy have a low score on fear of movement (r = -0.469,p<0.001). Mediation analysis showed that self efficacy was a significant mediator in the association between fear of movement and PA (Fig 3). The majority (73.2%) of the pathway between fear of movement and PA is explained by physical self-efficacy (Table 5).

**Table 5:** The relationship between fear of movement and PA is mediated by self-efficacy for 73.2%.

	Beta [95% CI]	Proportion mediated (%)
Indirect effect	-6.422 [-8.86;-4.23]	73.2%
Total effect	-8.775 [-12.42;-5.39]	

## Discussion

The present study, is to our knowledge, the first study which assesses fear of movement as a barrier to PA after renal transplantation. Important independent variables associated with fear of movement are history of MI, TIA and CVA and physical self-efficacy. Our results indicate that fear of movement is associated with low PA levels after renal transplantation. Regression analyses showed that a large part of the association of fear of movement with PA level (73%) is explained by a low level of self-efficacy.

We anticipated that the presence of fear of movement after renal transplantation has a negative effect on daily PA level resulting in a vicious cycle of inactivity. Indeed, among RTR with a low score for fear of movement, 54% met the guidelines for PA, compared to only 38% of the RTR with high fear of movement. With regard to the various domains of PA, we found that fear of movement was related to leisure time PA, sports and PA from occupational activities. From previous research we know that participation in social leisure time activities and sports is low in RTR [49]. Our results with regard to sports-and leisure time PA show that fear of movement might be an important underlying factor in these low participation rates. Fear of movement is also related to occupational PA. Accordingly, we found lower employment rates in the group with high fear of movement (27% versus 42% in the group with low fear of movement). These results suggest that RTR with high fear of movement scores are hindered in participation of activities which require a certain level of physical health and fitness.

The TSK-11 is originally designed for patients with musculoskeletal pain. Although, it is increasingly being used in other populations, thus, leading to the important issue, of whether clinical interpretation of TSK-11 is accurate in other patient groups like our renal transplant population. As the TSK-11 provides no specific information on the nature of the fear, it can be presumed that the nature of fear in RTR differ from the other populations in which the TSK-11 was used. While the source of fear is completely different from patients with musculoskeletal pain, the same concept of activity avoidance applies. The most important source of distress is fear of losing the graft [15,16]. Consequently, RTR can become over protective with regard to their transplanted kidney. It may be that RTR are insufficiently informed about the advantages and opportunities of PA and exercise after renal transplantation, leading to misconceptions and insecurity. This insecurity can lead to a low physical self-efficacy and to less PA, which could result in a viscous cycle of inactivity.

Regular PA is a preventive measure for CVD in the general population [6,7,9]. At the same time, CVD is the primary cause of death among kidney transplant recipients, and almost 50% of post-transplant deaths are attributable to CVD [5,7]. We previously found a strong relationship between low PA levels in RTR and increased risk for cardiovascular and all-cause mortality [11]. To effectively target PA after transplantation, it is important to identify common barriers to PA. In the current study, we found that history of myocardial infarction, TIA and CVA are important determinants of fear of movement. Targeting fear of movement in lifestyle intervention programs could improve the success of these interventions. Post-transplant healthcare providers should focus on the self-care practice predictors such as: patients’ health beliefs and removing known barriers to self-care.

Physical self-efficacy is shown to be a predictor of the adoption and maintenance of PA, and closely linked to fear of movement. Therefore, self-efficacy could be an important factor underlying the relationship between fear of movement and physical activity. Interestingly, we found that physical self-efficacy to a large extent mediated the relation between fear of movement and PA in RTR. Thus, fear of movement affects PA not only directly, but also through its effects on physical self-efficacy. There are several factors that could influence physical self-efficacy and fear of movement after renal transplantation, as fear of movement is linked to illness perceptions and threat avoidance [50]. Due to the often long history of chronic kidney disease and dialysis treatment, RTR may generally have limited exercise tolerance [14]. In addition, pharmacologic treatment with corticosteroids and calcineurin inhibitors may contribute to muscle wasting and muscle dysfunction [51,52]. Besides these factors, overall medical status and presence of co-morbidities like diabetes and cardiovascular disease might influence the confidence or ability to be physically active in RTR. This is in line with our finding that history of MI, TIA and CVA are independently associated with fear of movement. During exercise, RTR may perceive certain signs or symptoms as health threats which may lead to PA avoidance, according to the Common Sense Model of Self-regulation [50].

The perceived health threat may cause fear and distress as an emotional response, thereby influencing self-efficacy [25,27]. The cognitive response may be that the perceived threat is detrimental for the allograft. Coping with the emotional and cognitive responses may lead to threat avoidance behavior, in this case, needlessly avoiding harmless physical activities. Examples of perceived health threats during PA may include fatigue and breathlessness [53,54]. Our findings complement a qualitative study shortly after renal transplantation which discusses important barriers to exercise such, as being afraid of hurting oneself from exercise, experiencing pain, and having an open incision [55]. Since our findings suggest that perceived health threats hinder healthy PA behavior, it may be an important target for intervention after renal transplantation. Potentially helpful behaviour change techniques to overcome this barrier may include providing information about the health consequences of regular PA, and providing information about the behavior-health link. This might help to ameliorate the emotional (fear) and cognitive (PA avoidance) responses to perceived health threats [16].

Ample evidence underlines the importance of physical self-efficacy in PA interventions [23,25,26,28]. Bandura distinguishes four major sources that contribute to the development of self-efficacy; performance accomplishments, verbal persuasion, vicarious learning and physiological and emotional states [27]. Performance accomplishments is the most powerful source of self-efficacy because it is based on personal mastery experiences, with successful experiences leading to greater feelings of self-efficacy [27]. One of the most reliable behaviour change techniques for enhancing performance accomplishment is 'action planning’ (i.e. prompt detailed planning of the physical activity behaviour, such as what to do where and when) [56–58]. Action planning may help to attain physical activity goals, thereby increasing mastery experience [59]. Focusing on small successes that have been achieved enhances self-efficacy [27,59]. Verbal persuasion, like encouragement and constructive feedback, is the next source of self-efficacy. An eligible behavior change technique for improving this source of self-efficacy is 'verbal persuasion about the capacity'. For example when a physiotherapist, encourages and convinces a patient to perform a certain physical activity task, that patients feels more capable of performing the task. Another important source of self-efficacy is vicarious learning. Observing other persons that are similar to yourself succeed can increase your beliefs that you can perform the same task [56,59]. A behaviour change technique that can be used to foster this source of self-efficacy is 'social comparison'. For example, if physical activity was performed with RTR whom had higher levels of physical self-efficacy then this may act as vicarious learning. The last source contributing to self-efficacy is physiological and emotional states. Moods, emotions, fears and physiological symptoms like muscle pain and fatigue influence how one feels about their personal ability to perform PA. Strategies to increase this source of self-efficacy may include providing information about the health consequences of regular PA and reducing negative emotions like anxiety [27,56,59]. Recommendations for participating in PA after renal transplantation may include supervised exercise which would allow participants to understand what physiological symptoms are normal to experience whilst being active in a safe and supported environment to reduce fear. Future studies promoting physical activity in RTR should incorporate these behaviour change techniques to improve self-efficacy and target fear of movement.

This study has several strengths and limitations. Strengths of our study include the large sample size and the novelty of our findings in this population. Extensive data collection, including data on mental health, various domains of PA and data from twenty-four hour urine samples resulted in a well-defined description of the cohort. Some methodological topics warrant consideration. Our study is very heterogeneous, with variable time post transplant including only RTR from 1 year after renal transplantation. RTR in the early phase after renal transplantation might be facing other psychological factors, as they might still be recovering from their transplant operation. Future studies should investigate fear of movement in the early post transplant period. Another limitation of our study is that we only have baseline measurements of fear of movement, self-efficacy and physical activity. Fear of movement and self-efficacy are dynamic concepts which could vary over time and physical activity patterns could have been changed over time. Furthermore in this study PA was estimated by questionnaires, so recall bias and social desirability bias may influence internal validation. Objective measures such as exercise tolerance testing or use of accelerometers would have been more suitable. Nevertheless, questionnaires are valuable instruments to estimate PA in large epidemiological studies. The present study is observational in design with single measurements which makes it difficult to conclude on causality. For example future intervention studies should target fear of movement and physical self-efficacy, and study the effects on PA levels after transplantation. Finally, our study is limited by the long period of time since data collection and publication.

In summary, this study is the first to show the association of fear of movement with low levels of PA in RTR. A major part of this relationship was mediated by physical self-efficacy. Fear of movement and physical self-efficacy are important psychological factors which are likely to act as barriers to engage in a PA lifestyle. Clinical decision making in post-transplant care concerning PA should offer strategies to overcome these mental barriers to optimize the success of PA interventions after transplantation.
